# Z-ligustilide and anti-inflammatory prostaglandins have common biological properties in macrophages and leukocytes

**DOI:** 10.1186/s12986-018-0239-1

**Published:** 2018-01-16

**Authors:** Joseph Schwager, Lidia Gagno, Nathalie Richard, Werner Simon, Peter Weber, Igor Bendik

**Affiliations:** DSM Nutritional Products Ltd., Department of Human Nutrition & Health, P.O. Box 2676, CH-4002 Basel, Switzerland

**Keywords:** Chemokines, Cytokines, Inflammation, Macrophages, Z-ligustilide, NF-κB pathway, Anti-inflammatory prostaglandins, Peripheral blood leukocytes, THP-1 human monocytic leukemia cells

## Abstract

**Background:**

During inflammation, immune cells produce cytokines, chemokines and prostaglandins. This results in acute or chronic inflammation, which favor the development of degenerative diseases such as diabetes, obesity or cardiovascular diseases. Inflammatory processes are modulated by intrinsic and external factors. External factors are supposed to act via similar modes of action as do endogenous molecules and mediators. Both endogenous ligands and nutrient-derived metabolites might modify the extent and status of the cellular and systemic response during inflammation. Therefore, the biological activity of endogenous mediators was compared with nutrition-derived substances.

**Methods:**

Murine macrophages (RAW264.7 cells), in vitro differentiated human promyeloid THP-1 cells and peripheral blood leukocytes (PBL) were stimulated with LPS in the presence of z-ligustilide (LIG) or the endogenous PPARγ ligand 15deoxyΔ12,14-prostaglandin J_2_ (15d–PGJ_2_). Secretion of mediators of inflammation was measured by EIA, the Griess reaction and multiplex ELISA (Luminex®). Gene expression was quantified by real-time PCR. Nuclear translocation of NF-κB was measured by cytometric techniques.

**Results:**

LPS-activated RAW264.7 cells produced nitric oxide (NO), COX2-dependent prostaglandin E_2_ (PGE_2_), interleukins and chemokines. LIG concentration-dependently reduced the production of nitric oxide (NO) and PGE_2_, although it did not match the inhibitory potential of 15d–PGJ_2_. LIG inhibited the secretion of cytokines (IL-1α, IL-6, TNF-α) and differentiation factors (GM-CSF) in murine macrophages. It blunted the production of CCL2/MCP-1, but did not alter the secretion of CCL5/RANTES. LIG reduced mRNA levels of pro-inflammatory cytokines (e.g. TNF-α, IL-1α, IL-6), chemokines (CCL4/MIP-1β), and pro-inflammatory enzymes (iNOS). Similarly, LIG robustly impaired inflammatory mediators (e.g. CCL2/MCP-1, CCL3-MIP-1α, CCL4/MIP-1β, CXCL10/IP-10, IL-12p70, TNF-α) of LPS-activated human THP-1 cells and PBLs. Unexpectedly, it augmented the production of IL-1β, IL-6 and GM-CSF in PBLs.

**Conclusions:**

LIG diminished the extent of the inflammatory response measured by the production of different mediators or metabolites (NO, PGE_2_, interleukins, cytokines, chemokines). LIG acted at the transcriptional level and targeted the NF-κB signaling pathway. Since LIG and the anti-inflammatory prostaglandin 15d–PGJ_2_ share most of the analyzed biological features, we infer that they have similar modes of action. Hence, LIG acts as an anti-inflammatory prostaglandin and modulates cytokine- and chemokine-dependent inflammatory responses.

## Background

Inflammatory processes are involved in the etiology of different diseases such as atherosclerosis, diabetes or arthritis. Acute inflammation encompasses distinct phases - initiation, progression and resorption - which are tightly controlled by mediators [1]. Inflammatory stimuli provoke a rapid release of mediators, which trigger the onset of the inflammatory response. Activation and recruitment of cell populations such as macrophages/monocytes or neutrophils are major hallmarks of the systemic response. Eventually, acute inflammation is self-controlled and relies partly on mechanisms similar to those operational during progression [2, 3]. Conversely, in chronic inflammation some of these events are not contained and provoke prolonged production of mediators with concomitant tissue erosion and pain.

At the molecular level, various transcription factors including NF-κB and nuclear receptors are involved in the control of acute and chronic inflammation. Peroxisome proliferator-activated receptors (PPAR) regulate inflammatory responses of macrophages; thus natural PPARγ ligands modulate macrophage activation [4–6]. Endogenous PPARγ ligands are derived from polyunsaturated fatty acids and generated through the action of lipoxygenase and prostaglandin synthase [7, 8]. Oxidized low-density lipoprotein (ox-LDL) and cyclopentenone prostaglandins including 15-deoxyΔ12,14-prostaglandin J_2_ (15d–PGJ_2_) were identified as PPARγ ligands with pro-atherogenic and anti-inflammatory properties, respectively [9, 10]. Several studies demonstrated that PPARγ-dependent and PPARγ-independent mechanisms regulate inflammatory processes [11–15].

This study aimed at identifying natural substances that modulate inflammatory processes and therefore might be of use in preventing associated diseases. Secondary plant metabolites such as EGCG [16] and resveratrol [17, 18] were found to bind to PPAR subtypes and thus modulate inflammatory responses. Z-ligustilide (LIG), a phtalide isolated from *Ligusticum chuanxiong* has anti-diabetic and anti-inflammatory properties [19–22] and was identified as putative novel PPARγ ligand [23]. This prompted us to compare the nutrient-based substance and the endogenously produced anti-inflammatory 15d–PGJ_2_. The data demonstrate that LIG and 15d–PGJ_2_ share numerous features in modulating the cellular response to inflammatory stimuli and thus confer LIG the properties of an anti-inflammatory prostaglandin.

## Methods

### Reagents

**Fig. 1:**
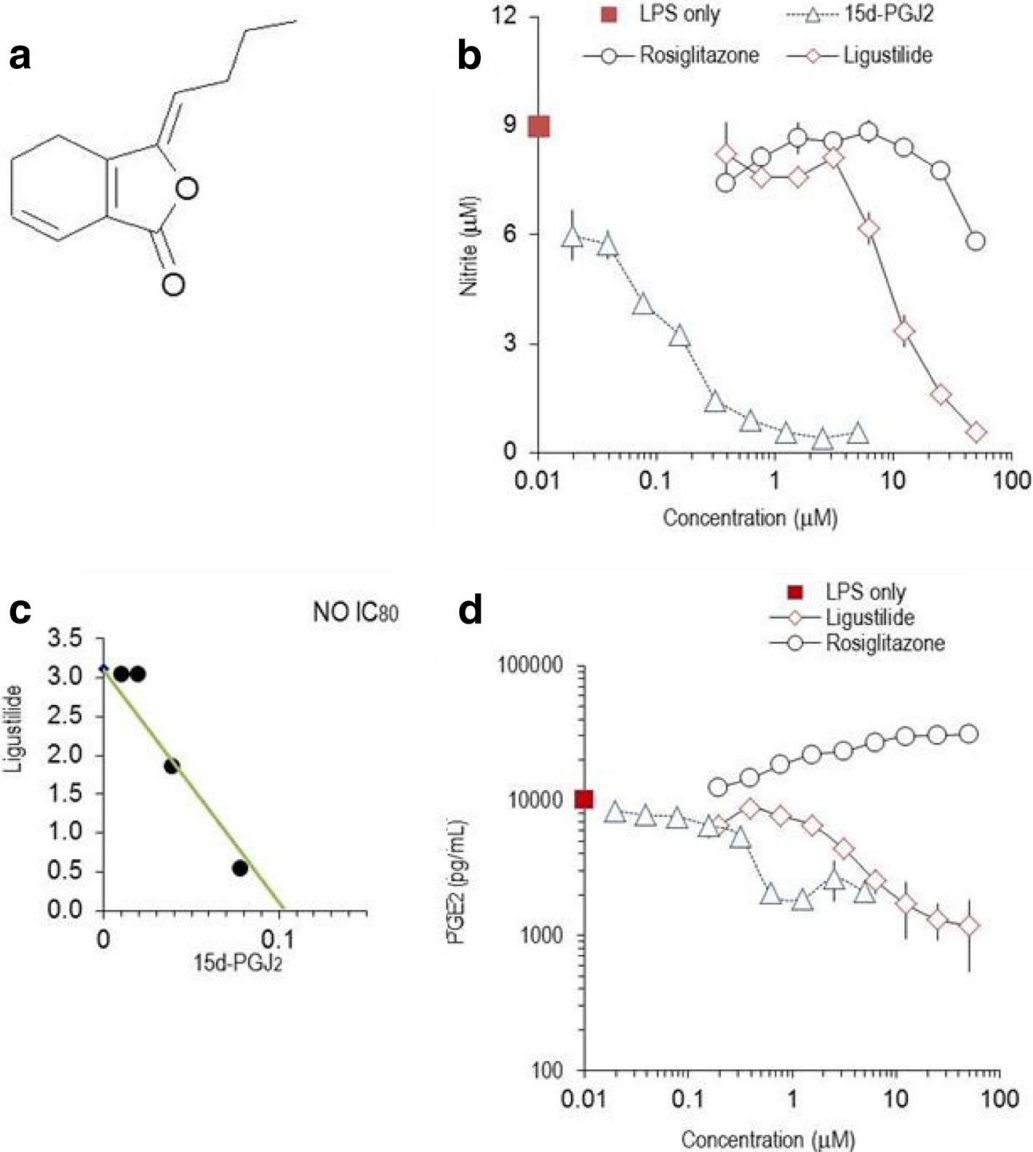
Z-ligustilide and 15d–PGJ_2_ alter nitric oxide and PGE_2_ in LPS-activated RAW264.7 cells. **a**: Molecular structure of z-ligustilide. **b**: Effect of z-ligustilide, 15d–PGJ2 and rosiglitazone on the production of nitric oxide (NO, measured as nitrite) by LPS-stimulated RAW264.7 cells, which were cultured for 24 h. Mean values (± SEM) of triplicate cultures from three independent experimental series are shown. **c**: Interactions between z-ligustilide and 15d–PGJ2 displayed in an isobologram (for details see reference [26] and Materials and Methods). A straight line was drawn between the IC_80_ value for z-ligustilide (in the absence of 15d–PGJ_2_) and the IC_80_ value of 15d–PGJ_2_ (in the absence of z-ligustilide). The values of the substances in combination fall on the straight line and thus reflect additive effects between z-ligustilide and 15d–PGJ_2_. **d**: Inhibition of PGE_2_ production by 15d–PGJ_2_, z-ligustilide and rosiglitazone in LPS-stimulated RAW264.7 cells which were cultured for 24 h. PGE_2_ was measured by EIA. Mean values ± SD of triplicates are shown. Similar results were obtained in 3 independent experimental series. Note the logarithmic scale of the axes

LIG was from MicroSource (Gaylordsville, CT), or was prepared by DSM Nutritional Products; it was also isolated from *Ligusticum chuanxiong* by Kieselgur column fractionation (Fig. 1a). Its purity was >98% (according to the manufacturer’s data sheet). 15-deoxyΔ12,14 prostaglandin J_2_ (15d–PGJ_2_) was from Cayman Chemicals (Ann Harbor, MI). Rosiglitazone was purchased from Shanco International, Inc. (Hazlet, NJ). L-NAME (L-N^G^-Nitroarginine methyl ester) was from Sigma, Saint-Louis, MO. Compounds were dissolved in DMSO and added to the culture medium concomitantly with the stimulus. Final DMSO concentration was 0.5%. Lipopolysaccharide (LPS, *E. coli* serotype 055:B5) and foetal bovine serum (FBS) were from Sigma. Ficoll-Isopaque was from Nycomed Pharma AS (Oslo, Norway). DMEM, RPMI Medium 1640, PBS, non-essential amino acids (NEAA), β-mercaptoethanol were from Invitrogen (Carlsbad, CA). Human recombinant interferon-γ (IFN-γ) was from Preprotech EC (London, UK). Primers and probes used in RT-PCR were designed with the Primer Express™ program (Applied Biosystems Inc., Foster City, CA;) and synthesized by Sigma.

### Cell culture

RAW264.7 cells were from ATCC (Manassas, VA) and cultured in DMEM supplemented with 10% FBS, 50 units/mL penicillin, 50 μg/mL streptomycin, L-glutamine and non-essential amino acids. Cells were used between passage 10 and 30. For experiments, cells were seeded into 6-well, 12-well or 96-well plates at 2, 1 and 0.05 × 10^6^ cells per well, respectively, and used after 2 days of pre-culture. Cells were starved in complete DMEM medium containing 0.25% FBS 18 h before the treatment. Cells were stimulated with LPS (1 μg/mL) for 4–24 h in phenol-free DMEM containing 0.25% FBS.

THP-1 cells (obtained from ATCC) were cultured in RPMI 1640 medium supplemented with 10% FBS, 50 units/mL penicillin, 50 μg/mL streptomycin, NEAA and 2 × 10^−5^ M β-mercaptoethanol. Cells were treated with 50 nM phorbol myristate acetate for 3 days. Cells were starved overnight in medium containing 0.25% FBS before being treated. Cells were stimulated with LPS (1 μg/mL) for 2–24 h in phenol-free RPMI containing 0.25% FBS.

Blood was obtained from healthy human volunteers. Peripheral blood leukocytes (PBLs) were also isolated from buffy coats obtained from local blood transfusion centers, using the Dextran sedimentation method to remove erythrocytes. Peripheral blood mononuclear cells were isolated by Ficoll-Isopaque gradient centrifugation and cultured in RPMI 1640 medium, supplemented with 0.25% FBS, NEAA, penicillin / streptomycin (50 U/mL / 50 μg/mL), and 5 × 10^−5^ M β-mercaptoethanol. Cell viability was determined by the Trypan Blue exclusion test and exceeded 95%. For in vitro cultures, cells were adjusted to 1 × 10^6^ cells/mL. Peripheral blood leukocytes were stimulated with LPS (1 μg/mL) and IFN-γ (20 U/mL) for 2–24 h in phenol-free RPMI containing 0.25% FBS.

### Measurements of cell viability

LDH was determined in supernatants from cell cultures immediately after harvesting, using a commercially available cytotoxicity kit (Promega, Madison, WI).

### Measurement of production of PGE_2_ and NO

Concentrations of nitrite, which is generated from cell-released nitric oxide, were determined by the Griess reaction [24]. PGE_2_ was quantified by EIA [25] according to the manufacturer’s instructions (Cayman Chemicals). All determinations were done in duplicates and at various dilutions of the culture supernatants.

### Isobolographic analysis of interaction between substances

The interactions between substances were evaluated using isobologram analysis essentially as described [26]: LIG and 15-PGJ_2_ were mixed at a fixed ratio, added to RAW264.7 cells at a large concentration range and their effects on LPS-induced NO and PGE_2_ production determined and expressed as IC_50_; concomitantly, effect of each individual substance was measured. Alternatively, to cultures containing a given concentration of LIG, 15d–PGJ_2_ was added at varying concentrations and the IC_50_ or IC_80_ was computed for the mixture of substances. The IC_80_ values were then used to create isobolograms as described [26, 27]. Synergistic effects were also computed with the CalcuSyn Version 2.0 software (Biosoft, Ferguson, MO).

### Determination of cytokine and chemokine production

Multiparametric kits were obtained from BIO-RAD Laboratories (Hercules, CA) and used in the LiquiChip Workstation IS200 (Qiagen, Hilden, Germany) according to the manufacturers’ instructions. We used the Bio-Plex Mouse Cytokine 23-Plex Panel and the Bio-Plex Human Group I cytokine Broad Range 27-Plex Panel; the data were acquired with the Luminex IS 2.3 software and evaluated with the LiquiChip Analyser software provided by Qiagen.

### Gene expression analysis

The isolation of total RNA, reverse transcription and quantitative real-time PCR have been done as described before [28]. The parameters for quantitative PCR and the calculation of fold changes (i.e. the relative expression of genes) are given in ‚Gene Expression Analysis Using Taqman Assays’ (http://www.thermofisher.com). Expression of 18S RNA was used as internal control (housekeeping gene). Expression values were normalized on the basis of unstimulated cells (where the threshold value is set as 1) and computed as fold changes (based on 2^-X^, where X returns to threshold value C_t_ of stimulated cells – threshold value C_t_ of unstimulated cells). Primer and probe sequences are given in Additional file 1: Table S1.

### Measurements of cytoplasmic/nuclear location of NF-κB

Cells were grown in 96-well plates and pre-incubated with various concentrations of LIG for 1 h. Cells were activated with LPS (1 μg/mL) for 20 min. Thereafter, cells were washed, fixed and permeabilized as detailed by Ding et al. [29]. Immunostaining for NF-κBp65 was performed using the Cellomics NF-κB Activation HitKitTM (Thermofisher Scientific Inc., Pittsburgh, PA). Nuclei were counter-stained with Hoechst dye. Immunofluorescence was measured by quantitative cytometric technique, ArrayscanTM, with the Cellomics instrumentation (Cellomics™ Inc.) expressed as Mean_CircRingAvgInten (for detail see: Cellomics HCS application guide). All treatments were done in triplicates.

### Statistical analysis

Data were evaluated by statistical tools described previously [28, 30, 31]. A *p* value <0.05 (calculated by using Student’s t test or one-way ANOVA) was considered to reflect statistically significant differences. Where appropriate, the Tuckey post-hoc test was applied for multiple comparisons. Statistical analysis was conducted with the SPSS software package, version 23.0.0. (SPSS, Munich, Germany).

## Results

### Z-ligustilide inhibits the production of nitric oxide and PGE2 in murine RAW264.7 cells

Macrophages respond to inflammatory stimuli by the exuberant secretion of cytokines, chemokines and other inflammatory mediators or enzymes. LPS-stimulated RAW264.7 cells produced significant quantities of nitric oxide (NO) and PGE_2_ within 24 h of culture [24, 25], whereas unstimulated cells produced >10-fold less mediators. LIG reduced NO production (Fig. 1b) with IC_50_ of 12.8 ± 1.4 μM (Table 1). L-nitroso-arginine-methyl ester (L-NAME) or resveratrol revealed to be less potent inhibitors with IC_50_ of 150 ± 12 μM and 28.5 ± 1.7 μM, respectively. 15d–PGJ_2_ abrogated NO production at >2 μM (IC_50_ 2.2 ± 0.6 μM). Rosiglitazone only marginally impaired NO production at concentrations >25 μM (Fig. 1b).

**Table 1:** IC_50_ values (in μM) for substances tested in LPS-activated RAW264.7 cells

	PGE_2_		Nitric Oxide	
	Mean ± S.E.M.	N	Mean ± S.E.M.	N
Z-ligustilide	9.3 ± 1.6	33	12.8 ± 1.4	58
15d–PGJ_2_	2.2 ± 0.5	16	2.3 ± 0.6	25
Rosiglitazone	>50	2	26.9 ± 1.9	16
L-NAME	>500	2	150 ± 12	2

Cells (in triplicates) were incubated with graded amounts of substances, stimulated with 1 μg/mL LPS and cultured for 24 h. PGE_2_ and NO were measured by EIA and the Griess reaction, respectively, and the IC_50_ values were calculated for each experimental series. N: number of independent experimental series. L-NAME: L-N^G^-Nitroarginine methyl ester

In order to test the hypothesis that substances might interact, we stimulated macrophages in the presence of combination of substances and visualized the effects on NO production by isobolographic analysis [26, 27]. LIG combined with 15d–PGJ_2_ led to inhibition curves, which reflected additive interaction as displayed in the isobologram (Fig. 1c). The points were on a straight line and thus indicated additive effects between the substances. Rosiglitazone did not reverse the inhibitory effect of LIG or 15d–PGJ_2_ nor was the effect of the combined compounds cumulative (not shown). Next, we explored the effect of LIG and 15d–PGJ_2_ on PGE_2_ production by LPS-simulated RAW264.7 cells. LIG (at >20 μM) abrogated PGE_2_ production (Fig. 1d), but it was a less potent inhibitor than 15d–PGJ_2_ (IC_50_ of 9.3 ± 1.6 μM and IC_50_ 2.2 ± 0.5 μM, respectively). When the two substances were combined and the inhibitory effect on PGE_2_ production analyzed, isobologram analysis revealed additive effects similar to those observed on NO production (data not shown). It should be noted that rosiglitazone concentration-dependently increased COX2-dependent PGE_2_ production (Fig. 1d). LIG and 15d–PGJ_2_ had only insignificant effects in unstimulated cells, which produced <10% of mediators secreted after LPS activation.

We further inspected whether the treatment of cells with compounds affected cell viability. LIG did not exert significant cytotoxic effects within a concentration range of <100 μM (Additional file 1: Figure S1) and was comparable to other micronutrients such as resveratrol or the catechin EGCG, which did not impair cell viability. Similarly, rosiglitazone or 15d–PGJ_2_ had no cytotoxic effects at concentrations <50 μM and <2.5 μM, respectively.

### Z-ligustilide modulates the inflammatory response in human THP-1 cells and peripheral blood leukocytes

**Fig. 2:**
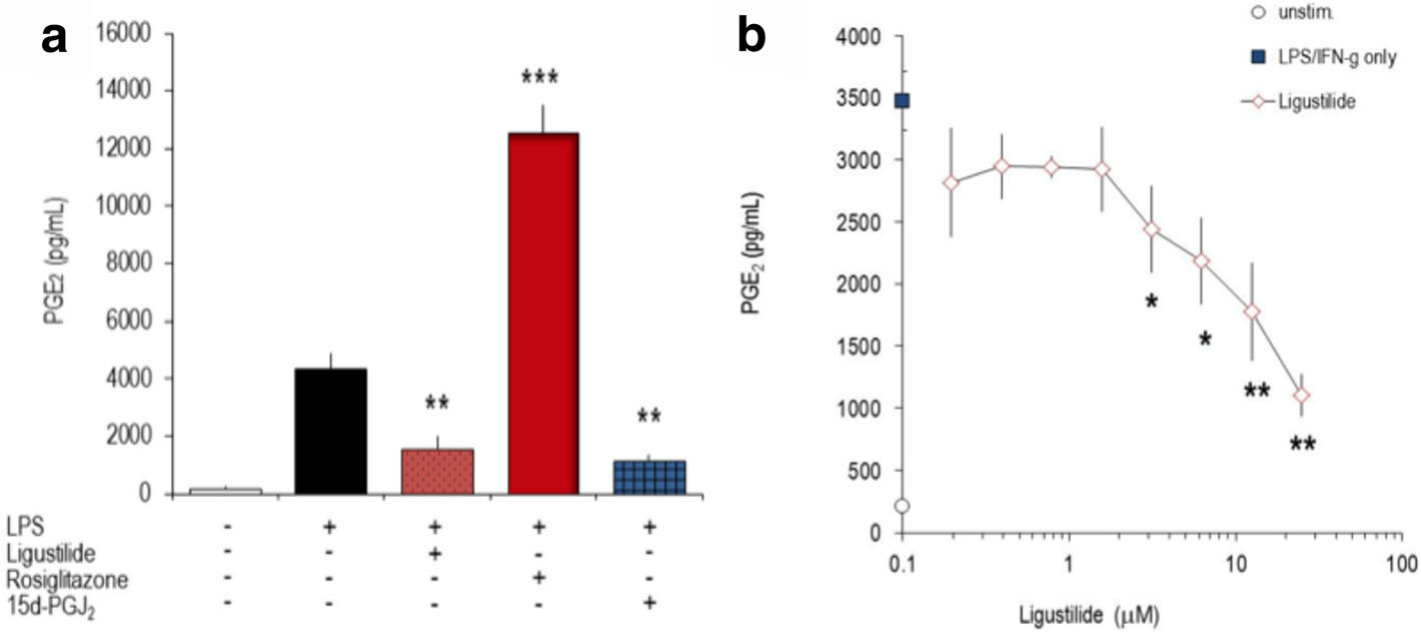
Z-ligustilide modulates PGE_2_ production in human THP-1 cells and PBLs. **a**: Effect of z-ligustilide, 15d–PGJ_2_ and rosiglitazone on THP-1 cells: Cells were differentiated in vitro with phorbol myristate acetate (20 nm/L) for 3 days, stimulated with LPS (1 μg/mL) in the absence or presence of z-ligustilide (25 μM), rosiglitazone (25 μM), or 15d–PGJ_2_ (2.5 μM). Secreted PGE_2_ was determined after 24 h by EIA. **b**: Freshly isolated peripheral blood leukocytes were stimulated with LPS/IFN-γ in the presence of graded amounts of z- ligustilide. Secreted PGE_2_ was determined after 24 h by EIA

We hypothesized that LIG modulated the production of inflammatory mediators in a similar way in different species. Therefore, THP-1 cells, a human monocytic leukemia cell line, were induced to differentiate in vitro, stimulated with LPS [32] and the effect of substances on eicosanoid synthesis was determined. LIG and 15d–PGJ_2_ blunted PGE_2_ production, whereas rosiglitazone augmented COX-2 dependent PGE_2_ levels in THP-1 cells (Fig. 2a). Likewise, human peripheral blood leukocytes (PBLs, consisting of mononuclear and polymorphonuclear cells) were stimulated with LPS/IFN-γ in the presence of various concentrations of LIG and the PGE_2_ secretion was determined. LIG dose-dependently decreased the inflammatory response as measured by the PGE_2_ production (Fig. 2b); 15d–PGJ_2_ had stronger inhibitory effects than LIG (not shown). Effects of LIG and 15d–PGJ_2_ on unstimulated cells were not significant. Collectively, murine macrophages and human cell lines or primary cells (PBLs) displayed a similar responsiveness to LIG.

### Z-ligustilide alters the production of cytokines, chemokines and differentiation factors secreted by murine macrophages and human peripheral blood leukocytes

**Fig. 3:**
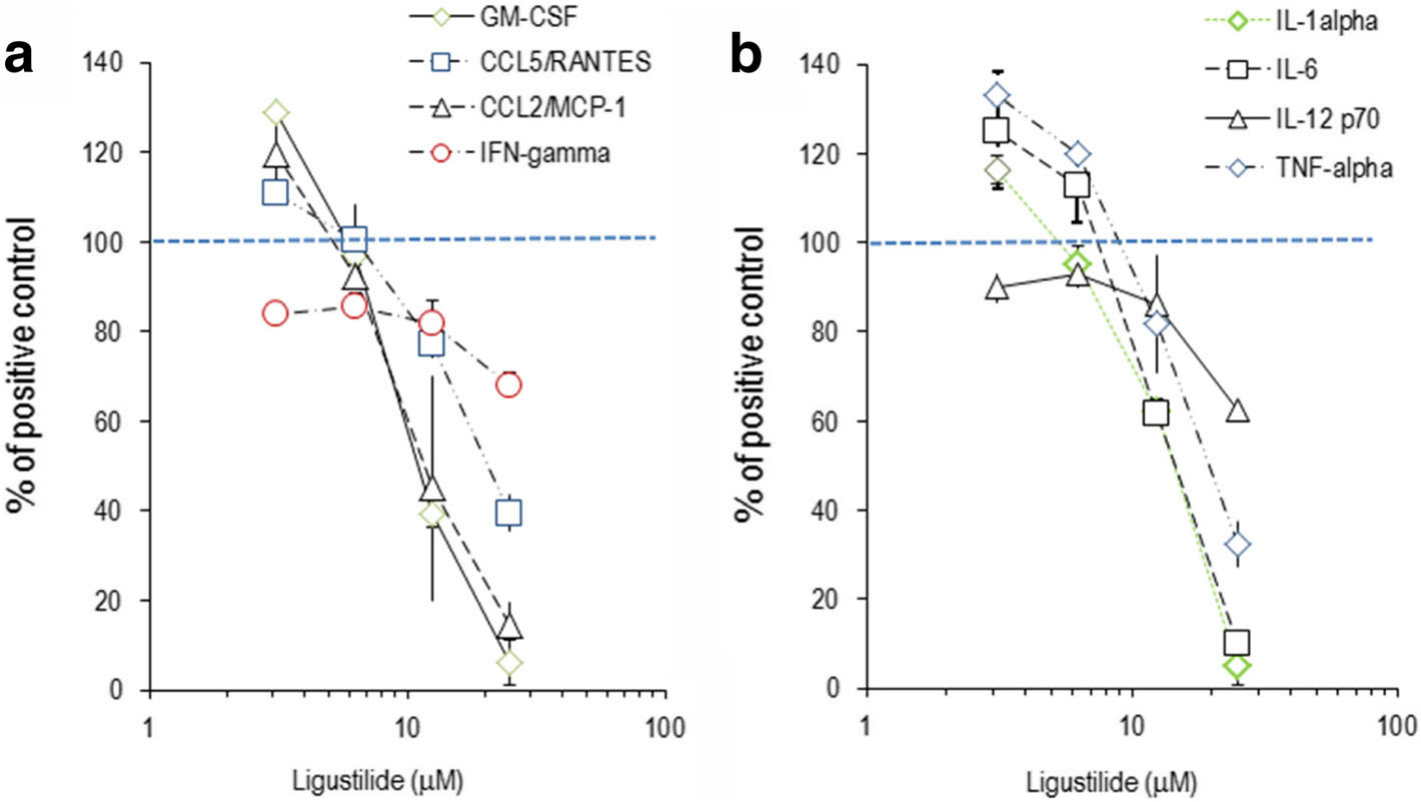
Production of cytokines and chemokines by RAW264.7 cells. RAW264.7 cells were stimulated for 24 h with LPS in the presence of indicated amounts of LIG. Secreted chemokines, cytokines and differentiation factors were measured by multiparametric analysis (i.e. GM-CSF, CCL5/RANTES,CCL2/MCP-1, IFN-gamma (in **a**); IL-1alpha, IL-6, IL-12p70, TNF-alpha (in **b**)). Data are expressed in % of the values obtained for LPS-stimulated cells and are the means (± CV) of triplicates from three independent experimental series. The straight dotted line indicates the level of mediators produced by ‘LPS alone’-treated cells. All values <80% of positive control were significantly lower (*p* < 0.01 [versus 100% positive control])

Next, we investigated the effect of LIG and 15d–PGJ_2_ on inflammatory proteins produced by murine macrophages. The secretion of 9 (of 14 measured) cytokines and chemokines was increased 4-fold to ~4000-fold in LPS-activated RAW264.7 cells. Among the analyzed differentiation factors and chemokines, LIG concentration-dependently impaired the secretion of GM-CSF, CCL2/MCP-1 and CCL4/MIP-1β; CCL5/RANTES was only affected by high phtalide concentrations (25 μM) (Table 2 and Fig. 3). It exerted similar inhibitory effects on IL-1α, IL-6 and TNF-α, whereas IFN-γ and IL-12p70 were virtually unaltered. 15d–PGJ_2_ shared with LIG all features of the inhibition pattern, except for GM-CSF, CCL4/MIP-1α, CCL5/RANTES and TNF-α (Table 2 and Fig. 3). It should be noted, however, that the two substances differed in their biological potency: on a stoichiometric basis, 15d–PGJ_2_ was ~ 10-fold more efficient than LIG.

**Table 2:** Inflammatory proteins secreted by RAW264.7 cells

*Protein*	*Secretion by unstimulated cells [pg/mL]*	*LPS-induced protein secretion* *(ratio LPS-stim./unstim.)*	*% inhibition by LIG (25 μM)*	*% inhibition by* *15-dPGJ*_*2*_ *(1.25 μM)*
CCL2/MCP-1	4005	65	86 ± 5 ^***)^	58 ± 39 ^**)^
CCL4/MIP-1β	39,250	7.0	40 ± 13 ^*)^	-9 ± 22
CCL5/RANTES	268	219	61 ± 4 ^**)^	2 ± 27
IFN-γ	22	4.2	32 ± 3 ^**)^	24 ± 4 ^*)^
GM-CSF	134	16	94 ± 5 ^**)^	91 ± 2 ^***)^
IL-1α	9	233	95 ± 4 ^***)^	50 ± 1 ^**)^
IL-6	28	1442	90 ± 0 ^***)^	76 ± 15 ^*)^
IL-12p70	84	4.6	38 ± 2 ^**)^	27 ± 7 ^*)^
TNF-α	40	3820	68 ± 5 ^**)^	67 ± 2 ^**)^

RAW264.7 cells were stimulated with LPS and cultured for 24 h in the presence of substances. Secreted proteins were measured by multiparametric analysis (Luminex technology). The LPS-induced increase of secreted proteins is given as ratio of values obtained in stimulated versus unstimulated cells. * *p* < 0.05; ** *p* < 0.01; *** *p* < 0.005 (‘LPS + substance’ treated versus ‘LPS-only’ treated cells)

**Fig. 4:**
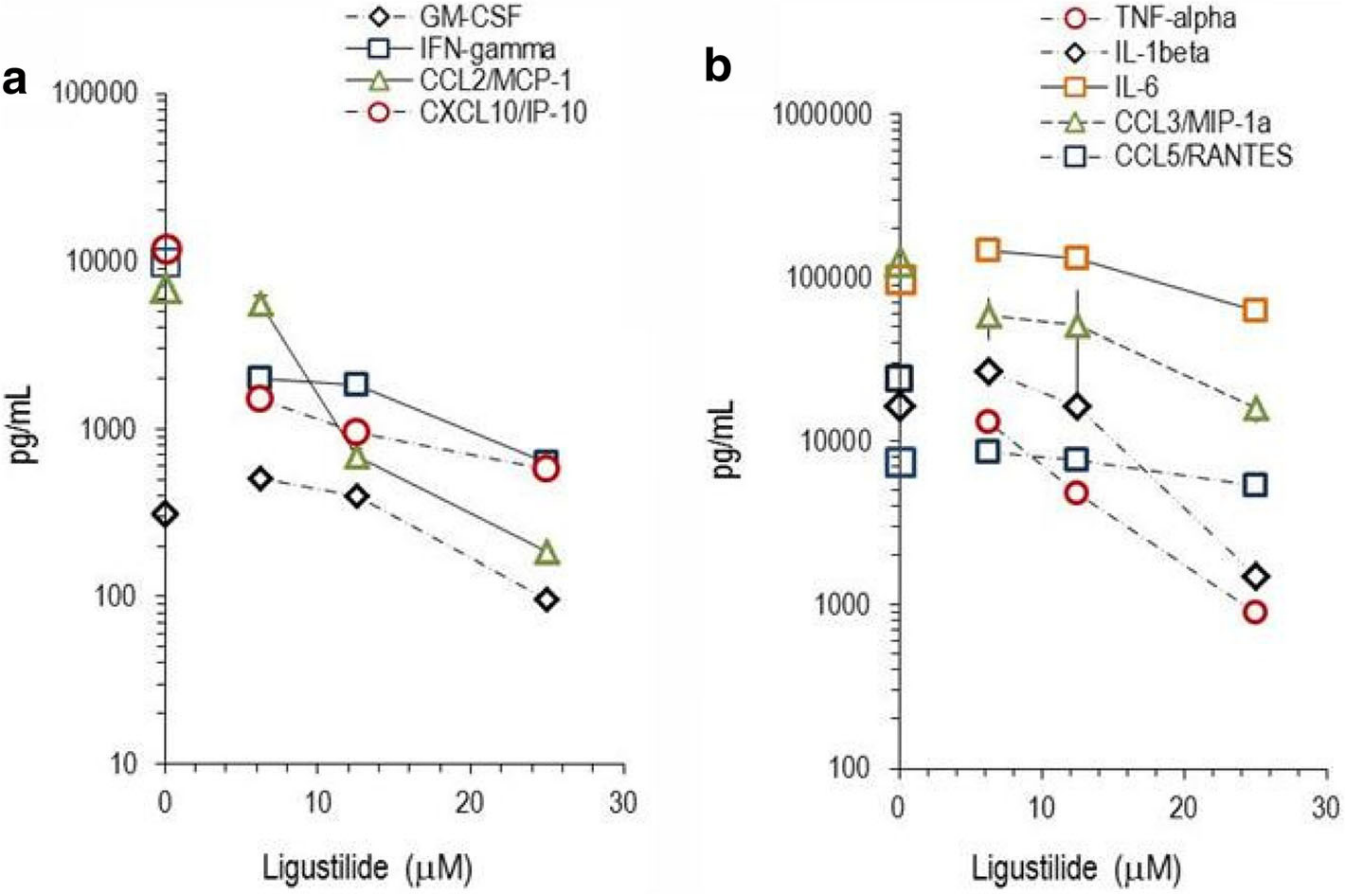
Production of cytokines and chemokines by PBLs. Freshly isolated peripheral blood leukocytes were stimulated for 24 h with LPS/INF-γ in the presence of indicated amounts of LIG. Secreted chemokines, cytokines and differentiation factors (i.e. GM-CSF, IFN-gamma, CCL2/MCP-1, CXCL10/IP-105/RANTES (in **a**); TNF-alpha, IL-1beta, IL-6, CCL3/MIP1alpha, CCL5/RANTES (in **b**)) were measured by multiparametric analysis and are indicated in pg/mL. ‘LPS/INF-γ alone’ values for the molecules are indicated on the y-axis. Data are means ± SD of triplicate cultures of PBLs

We extended this analysis to human PBLs, where the activation with LPS/IFN-γ led to >4-fold increased secretion of inflammatory proteins, which included chemokines (CCL3/MIP-3α, CXCL8/IL-8, CXCL10/IP-10) and cytokines (TNF-α, IL-1β, IL-6, IL-12p70). CCL5/RANTES and CCL2/MCP-1 were constituently expressed at high levels presumably by polymorphonuclear cells (PMNL), contained in PBLs. The secretion of GM-CSF, CCL2/MCP-1 and TNF-α was drastically decreased by raising concentrations of LIG (Fig. 4 and Table 3). Conversely, IFN-γ, CXCL/10IP-10, IL-1β, IL-6 and CCL5/RANTES were only marginally influenced. This further revealed that the phtalide specifically affected the secretion of distinct cytokines and chemokines. LIG and 15d–PGJ_2_ differed in two important aspects in their anti-inflammatory effects on PBLs: CCL2/MCP-1 and TNF-α production were not impaired by the cyclopentenone prostaglandin, whereas LIG strongly altered them (Fig. 4). It should be noted that GM-CSF and IL-6 secretion was markedly enhanced when LPS/INF-γ activated PBLs were treated with LIG or 15d–PGJ_2_. This was substantially more potent than LIG in its biological effects.

**Table 3:** Inflammatory proteins secreted by human PBLs

*Protein*	*Unstimulated cells 24 h) [pg/mL]*	*Protein secretion* *(ratio LPS-stim./ unstim.)*	*% inhibition by Lig (6.25 μM)*	*% inhibition by 15d–PGJ*_*2*_ *(1.25 μM)*
CCL2/MCP-1	8540	0.8	87 ± 3 ^**)^	- 37 ± 34
CCL3/MIP-1α	6055	20.6	61 ± 4 ^*)^	67 ± 36 ^**)^
CCL4/MIP-1β	35,000	7.0	68 ± 7 ^**)^	51 ± 14 ^*)^
CCL5/RANTES	9225	0.8	< 2	<2
CXCL8/IL-8	20,700	21.1	<2	<2
CXCL10/IP-10	529	22.6	89 ± 3 ^**)^	86 ± 12 ^**)^
IFN-γ	22	10.4	84 ± 1 ^**)^	85 ± 9 ^**)^
TNF-α	982	24.8	46 ± 4 ^*)^	−16 ± 18
IL-1β	1055	15.5	−57 ± 5 ^*)^	38 ± 0 ^*)^
IL-6	11,400	8.6	−51 ± 3 ^**)^	−135 ± 32 ^**)^
IL-12p70	12	21.1	89 ± 2 ^**)^	85 ± 12 ^**)^

Peripheral blood leukocytes were isolated and cultured for 24 h at the indicated treatments. The proteins secreted into the culture supernatants were measured by multiparametric analysis. * *p* < 0.05; ** *p* < 0.01; *** *p* < 0.005 (‘LPS + substance’ treated versus ‘LPS-only’ treated cells)

**Fig. 5:**
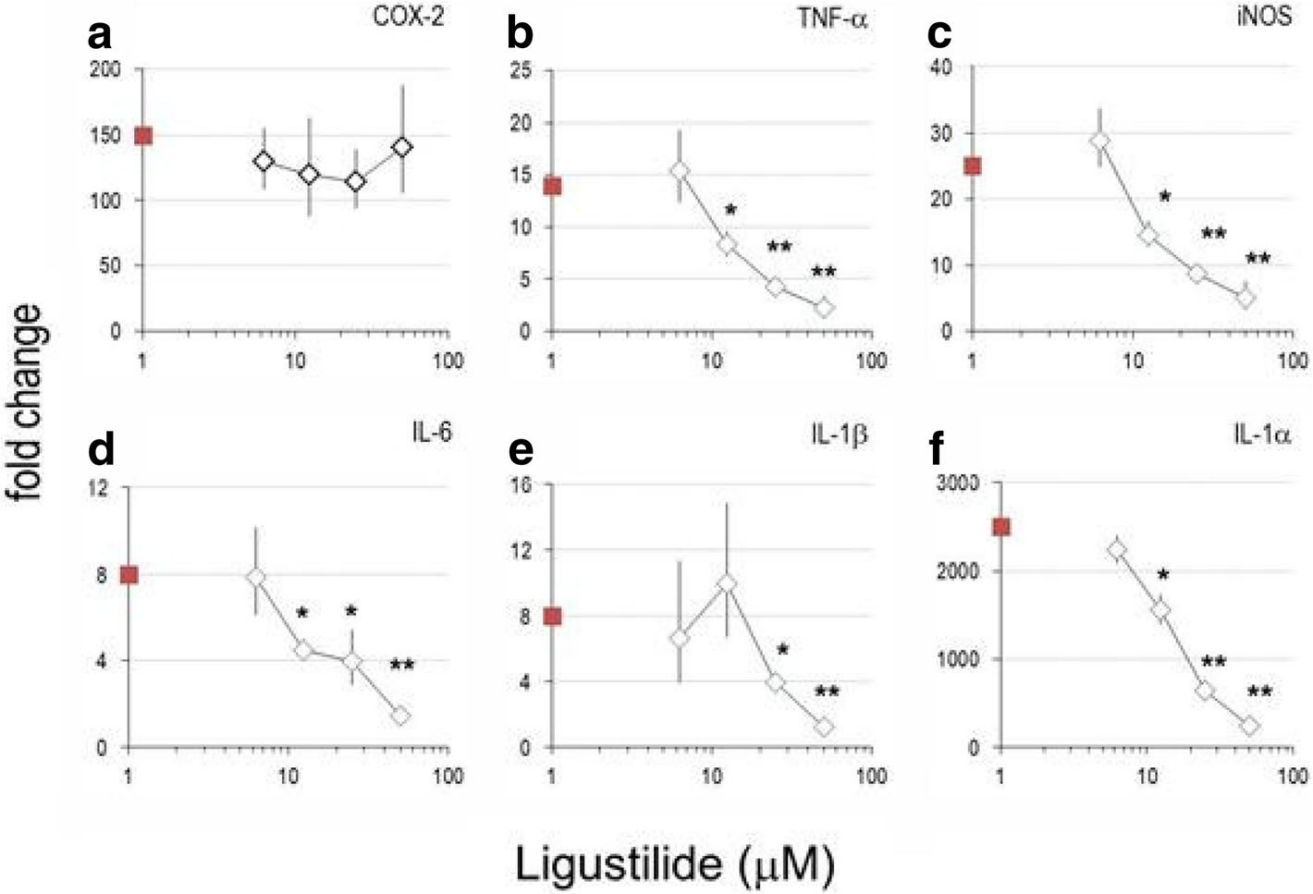
Effect of z-ligustilide on inflammatory genes expression of murine macrophages. Unstimulated or LPS-stimulated RAW264.7 cells were cultured for 4 h in the presence of graded amounts of LIG; mRNA levels were determined by RT-PCR. mRNA levels are indicated as fold change (y axis), which was calculated as indicated in Materials and Methods. Bars represent mean values of fold change +/− errors (see [28]) of triplicates (versus unstimulated cells). * *p* < 0.05; ** *p* < 0.01 (versus ‘LPS alone’ stimulated cells). **a**: COX-2; **b**: TNF-α; **c**: iNOS; **d**: IL-6; **e**: IL-1β; **f**: IL-1α

Using quantitative real-time PCR technology, we have evaluated the influence of LIG on inflammatory genes. Since macrophages drastically up-regulated inflammatory gene mRNA expression within 1–6 h following LPS-stimulation, we have chosen to analyze the influence of compounds in cells after 4 h of culture. There were notable differences in the basal expression levels in unstimulated macrophages with weakly (e.g. COX-2, iNOS, prostaglandin E synthase [PGES], IL-6, IL-1β, PPARγ1), moderately (e.g. COX-1, prostaglandin EP-2 receptor [EP-2], hematopoietic-type prostaglandin D synthase [PGDS], IL-1α) and abundantly expressed genes (e.g. TNF-α, fibronectin receptor-α [FNR-α], PPARβ, CCL4/MIP-1β) (data not shown). LPS induced a substantial increase of mRNA of interleukins and cytokines (e.g. IL-6, IL-1α and TNF-α) but also of iNOS and COX-2, whereas other genes were down-regulated (e.g. PGDS, EP-2, PPARγ1) (Fig. 5, Additional file 1: Figure S2). Unlike LIG, 15d–PGJ_2_ modulated PPARγ expression and shifted it towards pre-homeostatic/inflammatory levels. LIG reduced expression levels of iNOS, IL-1α, IL-1β, TNF-α and CCL4/MIP-1β by up to 90% in the concentration range of 6.25–50 μM. Interestingly, LPS-inducible COX-2 gene expression did not significantly change in the presence of LIG.

**Fig. 6:**
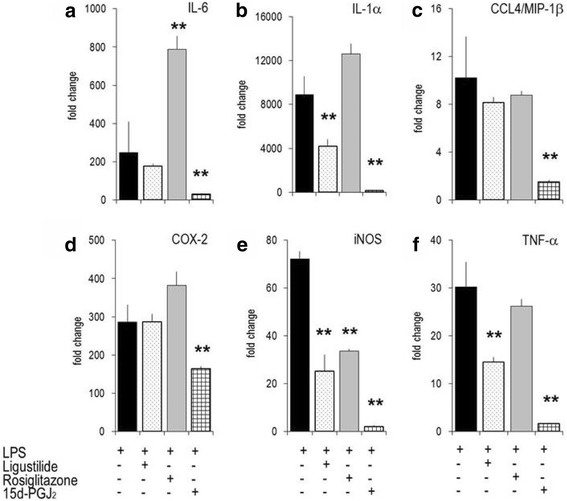
Modulation of gene expression by z-ligustilide, rosiglitazone and 15d–PGJ_2_ in RAW 264.7 cells. Unstimulated or LPS-stimulated RAW264.7 cells were cultured for 4 h in the presence of graded amounts of LIG (25 μM), rosiglitazone (25 μM) or 15d–PGJ_2_ (2.5 μM). mRNA levels were determined by RT-PCR. Bars represent mean values of fold change +/− errors of triplicates (versus unstimulated cells; set at 1). ** *p*-value <0.01 (versus ‘LPS-alone’ stimulated cells). **a**: IL-6; **b**: IL-1α; **c**: CCL4/MIP-1β; **d**: COX-2; **e**: iNOS; **f**: TNF-α

Since the expression of inflammatory genes in macrophages is in part PPARγ dependent [4, 5, 15], we compared the effects of PPARγ ligands like rosiglitazone or 15d–PGJ_2_ with LIG (Fig. 6). LIG (at 25 μM) significantly down-regulated 3 of 10 genes, while it up-regulated PPARβ and the receptor of PGE_2_ and EP-2 [33]. 15d–PGJ_2_ modulated inflammatory gene expression in a similar pattern, as did LIG, yet its effects were substantially stronger. Remarkably, it shifted expression of PPARγ1 and PGDS to base-line homeostasis (Additional file 1: Figure S2). Rosiglitazone shared some features with LIG (and thus also with 15d–PGJ_2_), but unlike LIG, it increased the expression of IL-1α, IL-6 and COX-2. Taken together, in murine macrophages the biological effects of LIG matched those of 15d–PGJ_2_ but differed from rosiglitazone.

### Z-ligustilide modulates expression of inflammatory genes in human THP-1 cells

**Fig. 7:**
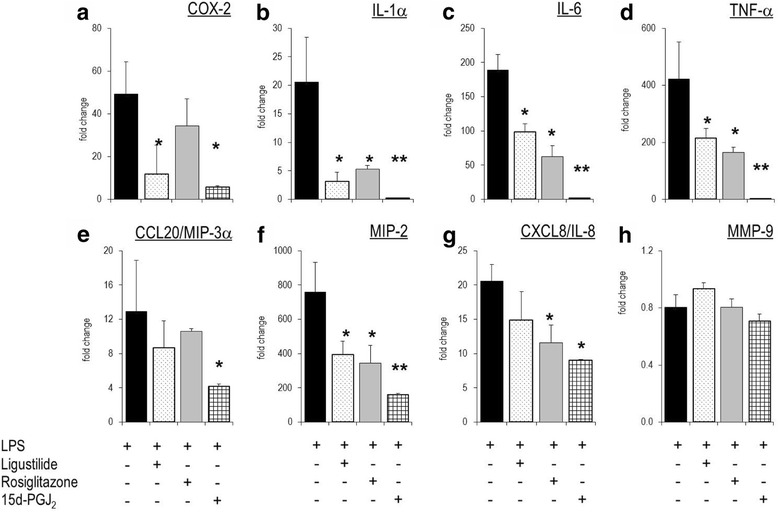
Effect of z-ligustilide on the expression of genes involved in the inflammatory response of THP-1 cells. Quantitative RT-PCR was made with RNA obtained from THP-1 cells that were stimulated with LPS for 4 h without or with LIG (25 μM), rosiglitazone (25 μM), 15d–PGJ_2_ (2.5 μM). **a**: COX-2; **b**: IL-1α; **c**: IL-6; **d**: TNF-α; **e**: CCL20/MIP-3α; **f**: MIP-2; **7 g**: CXCL8/IL-8; **h**: MMP-9. Bars represent mean values of fold change +/− errors of triplicates (versus unstimulated cells). * *p* < 0.05; ** *p* < 0.01 (versus ‘LPS-only’ stimulated cells)

Similar to murine macrophages, THP-1 cells responded to LPS stimulation by extensively up-regulated interleukins (IL-1α, IL-6), chemokines (IL-8, CXCL2/MIP-2, CCL20/MIP3α) or cytokines (e.g. TNF-α) (Fig. 7). All three compounds tested altered the gene expression pattern in a similar way, and their effects could be ranked as for RAW264.7 cells (i.e. 15d–PGJ_2_ > LIG > rosiglitazone). Other genes involved in inflammatory pathways including PPARα, 5-LOX and MMP − 9 were refractory to LIG, 15d–PGJ_2_ and rosiglitazone (data not shown).

### Z-ligustilide inhibits nuclear translocation of NF-κBp65

**Fig. 8:**
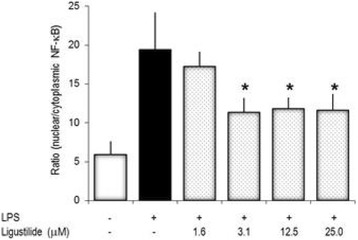
Early events in cell activation are impaired by z-ligustilide. RAW264.7 cells were pre-treated with LIG, activated with 1 μg/mL LPS for 20 min and the cytoplasmic-nuclear translocation of NF-κBp65 determined by quantitative cytometric technique. Y-axis: Ratio of nuclear versus cytoplasmic immunofluorescence. * *p* < 0.05 (relative to LPS-treated cells)

In order to further detect early effects of LIG on cell activation, the nuclear translocation of NF-κB during LPS-activation of RAW264.7 cells was measured by quantitative cytometric techniques. Cells responded to stimulation within 20 min by a substantial accumulation of NF-κBp65 in the nucleus; this was reflected by the shift in the ratio of nuclear/cytoplasmic fluorescence as shown in Fig. 8. In cells, which were pre-treated with LIG, the translocation of NF-κBp65 into the nucleus was significantly reduced in a concentration-dependent way.

## Discussion

The coordinated responses to inflammatory stimuli include synthesis and secretion of mediators during the progression of inflammation and its resolution by orchestrated production of endogenous molecules that terminate inflammation. Among these, cyclopentenone prostaglandins, lipoxins, NF-κB and mediators of apoptosis have recently attracted considerable interest (reviewed in [2]. Any deviation in these fine-tuned interactions can lead to an excessive and uncontrolled response that might result in chronic inflammatory processes. Given the fact that inflammatory responses are vital for the host’s appropriate defense against pathogens and external insults, the major challenge relies in the containment of inflammation i.e. its appropriate timing and extent of resolution. In this study, we have shown that LIG behaves to a large extent like anti-inflammatory prostaglandins and thus might contribute to the adequate resolution of inflammation as well as to the attenuation of chronic inflammatory processes (Additional file 1: Table S2).

Our experimental approach was instigated by the identification of LIG, a natural substance with anti-diabetic properties [23]. We hypothesized that LIG and PPARγ agonists like rosiglitazone interfere with inflammatory responses in a similar manner and compared their impact on hallmarks of inflammation including the gene expression and production of nitric oxide, PGE_2_, chemokines, interleukins and cytokines. Whereas rosiglitazone moderately impaired NO production and even enhanced PGE_2_ secretion, LIG potently diminished the production of both metabolites and performed like 15d–PGJ_2_. Previously, 15d–PGJ_2_ had been identified as a PPARγ ligand that induced adipogenesis [34, 35]. More recent data showed that PPARγ ligands regulated inflammatory responses by interfering with the expression of iNOS and cytokines including IL-1, TNF-α or gelatinase B (MMP-9) in macrophages, which express the respective PPAR isoform [4, 5]. Yet, cyclopentenone prostaglandins influenced the inflammatory responses via different signaling pathways in macrophages from PPARγ−/− mice [36, 37]. Conceivably, insufficient expression of PPAR isoforms in the studied cellular systems might account for the unresponsiveness to PPARγ ligands. Yet, PPARβ and PPARγ mRNA were readily detected in RAW264.7 cells, THP-1 cells and peripheral blood leukocytes (not shown). Collectively, the data support the notion that there is an association of LIG activity and PPARγ expression in macrophages; yet, this interaction is not causally related to inflammatory pathways [10, 38].

Experimental data provide a mechanistic explanation for PPARγ-independent suppression of NF-κB by 15d–PGJ_2_ [15, 39]. LIG and 15d–PGJ_2_ possess α, β or α, β, γ unsaturated carbonyl structures that react with nucleophiles including free sulfhydryl groups of glutathione or cysteine. This prevents the binding of p65 homodimers of the NF-κB complexes [15, 40] and subsequent gene activation. In the NF-κB signaling cascade, IκB-kinase also contains cysteine residues that are prone to covalent modification. Indeed, cyclopentenone prostaglandins specifically inhibit IκB degradation [15]. Importantly, since LIG and 15d–PGJ_2_ both have unsaturated carbonyl structures, they might share similar modes of action, as has been described for other natural substances [40, 41]. By structural analogy to 15d–PGJ_2_, there is evidence for a structure-activity relationship between LIG and the effect on NF-κB. LIG and 15d–PGJ_2_ might differ in their biological half-life and the propensity to bind to reactive groups, which would explain their different IC_50_ values. The observation that LIG reduced nuclear translocation of NF-κBp65 in LPS-stimulated cells provides additional evidence that it modulated cellular activities along the NF-κB signaling pathway. It should be noted that sensu stricto the immunofluoerescence data only show that LIG impaired the cytoplasmic-nuclear translocation of NF-κB.

The production of most of the cytokines and chemokines was blunted by LIG in macrophages, monocytic leukemia cells (THP-1) and PBLs. Notable exceptions were IL-1β and IL-6, which are assigned pro-inflammatory properties. LIG increased the expression of these ILs in the blood compartment, but not in macrophages or monocytic cells. We have made similar observations with other nutrients like ω-3 PUFAs, resveratrol and tomato aqueous extracts [28, 30, 31]. This emphasizes the dichotomic effects of nutrients in distinct cellular compartments. Conceivably, an enhanced expression of IL-6 and IL-1β ameliorate the adaptive immune response and might act on the differentiation of the M1 and M2 macrophage subtypes.

The biological consequences of this mode of action are far-reaching and give a plausible explanation for the observed pleiotropic activity pattern of LIG. The genes that were down-regulated by 15d–PGJ_2_ and LIG (i.e. IL-1α, IL-1β, IL-6, IL-8, TNF-α, iNOS, CCL4/MIP-1β) possess NF-κB regulatory sequences/binding sites or are susceptible to the NF-κB signalling pathway (see also [42, 43]. Transcriptional activation in response to inflammatory stimuli can further be mediated by combined action with other factors [44, 45]. Given the importance of NF-κB in inflammatory diseases (reviewed in e.g. [46], even minor shifts in the amount, cellular localization or association of NF-κB elements drastically influence the outcome of the response. Admittedly, NF-κB ablation is not a panacea for inflammation, since it can result in severe apoptotic tissue damage [47]. A notable exception is the COX-2 expression in RAW264.7 cells, which remains unaffected by LIG (Fig. 5), while PGE_2_ production was inhibited (Fig. 1b). Concomitantly, PGES expression decreased in LIG treated cells, whereas mRNA levels of COX-2, EP-2 and PGDS were maintained. Consequently, LIG appears not to affect the part of the eicosanoid synthesis pathway that is required for the resorption of inflammation. Preserved COX-2 expression, mediated by LIG, ought to be beneficial during the resorption of inflammation when COX-2 is required for the concurrent PGDS-dependent synthesis of cyclopentenone prostaglandin PGJ_2_ [48].

We have demonstrated in this study that LIG, and to a larger extent 15d–PGJ_2_, markedly diminished the secretion of chemokines such as CXCL8/IL-8 and macrophage inflammatory proteins, CCL4/MIP-3α. Quantitative RT-PCR analysis revealed that the compounds attenuated the expression of these and other chemokines (CXCL1/MIP-1, CXCL2/MIP-2) by activated cells of the macrophage lineage. The CXC chemokines (e.g. murine CXCL2/MIP-2, human CXCL8/IL-8) play a role in recruitment of neutrophils, while the CC chemokine CCL4/MIP-3α recruits macrophages and lymphocytes. Chemokines and cytokines are mutually induced by LPS in a specific temporal pattern with pro-inflammatory cytokines usually preceding chemokine expression. This cross-talk is controlled by NF-κB and relies on NF-κB consensus sequences in the promoter region of chemokine and cytokine genes [49–52]. Conceivably, LIG and related compounds have an indirect effect on chemokine expression e.g. through inhibition of TNF-α expression, which in turn affects chemokine activation. As a consequence of the versatile in vitro effects of LIG described in this study, we anticipate that LIG has in vivo activities in acute inflammation models. Indeed, LIG proved to attenuate inflammation in the carrageenan-induced paw edema model (D. Raederstorff, unpublished results). At this time, comparable studies done in human have not been published. Therefore, the effects on acute and chronic inflammation in humans needs to be established in appropriate nutritional intervention trials.

## Conclusions

LIG is a potent anti-inflammatory natural substance that modulates the inflammatory response at various levels. Both by its structure and activity profile it is closely related to the cyclopentenone prostaglandins 15d–PGJ_2_. This confers LIG the profile of an anti-inflammatory prostaglandin that regulates the resorption of acute inflammation and blunts chronic inflammation.

## Additional file

Additional file 1: Figure S1. Viability of RAW 264.7 cells after incubation with graded amounts of ligustilide, EGCG, resveratrol, 15d–PGJ2 and rosiglitazone for 24 h. **Figure S2.** Regulation of inflammatory gene expression in RAW264.7 cells by ligustilide (25 μM) rosiglitazone (25 μM) and 15d–PGJ2 (2.5 μM). **Table S1**. Synopsis of effects on protein secretion by (murine) macrophages or peripheral blood leukocytes (human). **Table S1.** Sequences of primers and probes used in quantitative real-time PCR. **Table S2.** Comparison of effects of PGJ_2_ and ligustilide on cytokines and chemokine expression. (DOCX 465 kb)

## Abbreviations

CCL: Ligand of the C-C family of chemokines
CXCL: Ligand of the C-X-C family of chemokines
IC_50_: 50% (half-maximal) inhibitory concentration
IC_80_: 80% maximal inhibitor concentration
IL: Interleukin
INF: Interferon
iNOS: inducible nitric oxide synthase
LIG: Ligustilde
LPS: Lipopolysaccharide
NF-κB: Nuclear factor-kappaB
PGE_2_: Prostaglandin E_2_
PGJ_2_: Prostaglandin J_2_
PPAR: Peroxisome proliferator-activator receptor
TNF-α: Tumor necrosis factor-α
