# Supplementary material for: Z-ligustilide and anti-inflammatory prostaglandins have common biological properties in macrophages and leukocytes
Source: Nutr Metab (Lond). 2018 Jan 16;15:4. doi: 10.1186/s12986-018-0239-1 (PMC5771029; doi:10.1186/s12986-018-0239-1)
Supplement: Additional file 1: Figure S1. — Viability of RAW 264.7 cells after incubation with graded amounts of ligustilide, EGCG, resveratrol, 15d–PGJ2 and rosiglitazone for 24 h. Figure S2. Regulation of inflammatory gene expression in RAW264.7 cells by ligustilide (25 μM) rosiglitazone (25 μM) and 15d–PGJ2 (2.5 μM). Table S1. Synopsis of effects on protein secretion by (murine) macrophages or peripheral blood leukocytes (human). Table S1. Sequences of primers and probes used in quantitative real-time PCR. Table S2. Comparison of effects of PGJ2 and ligustilide on cytokines and chemokine expression. (DOCX 465 kb) [file 12986_2018_239_MOESM1_ESM.docx]

**Figure S1**: Viability of RAW 264.7 cells after incubation with graded amounts of ligustilide, EGCG, resveratrol, 15d-PGJ_2_ and rosiglitazone for 24 h. Only 15d-PGJ_2_ >25 mM markedly reduced cell viability as measured by the LDH release.

**
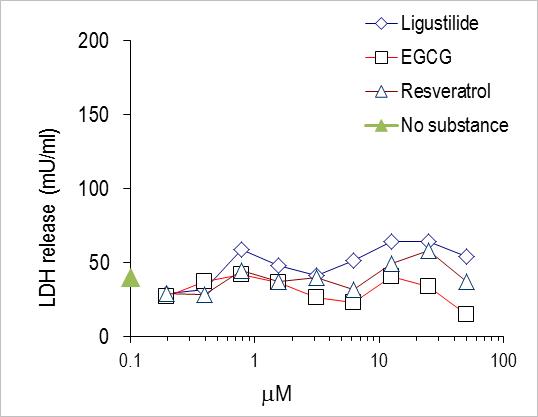
**


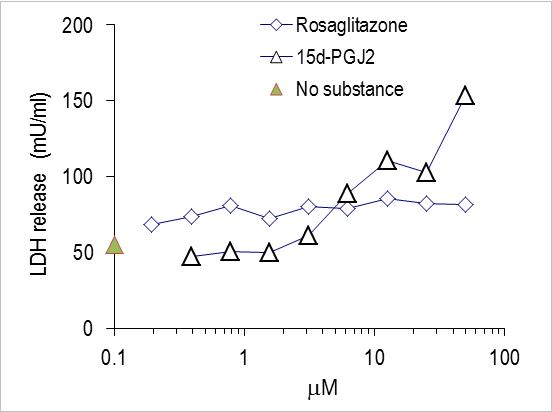


**Figure S2**: Regulation of inflammatory gene expression in RAW264.7 cells by ligustilide (25 μM) rosiglitazone (25 μM) and 15d-PGJ_2_ (2.5 μM).

PPAR-γ1: peroxisome proliferator activation receptor gamma1; EP2: eicosanoid receptor 2; PGDS: prostglandine D synthase, FNR-α: fibronectin receptor-alpha


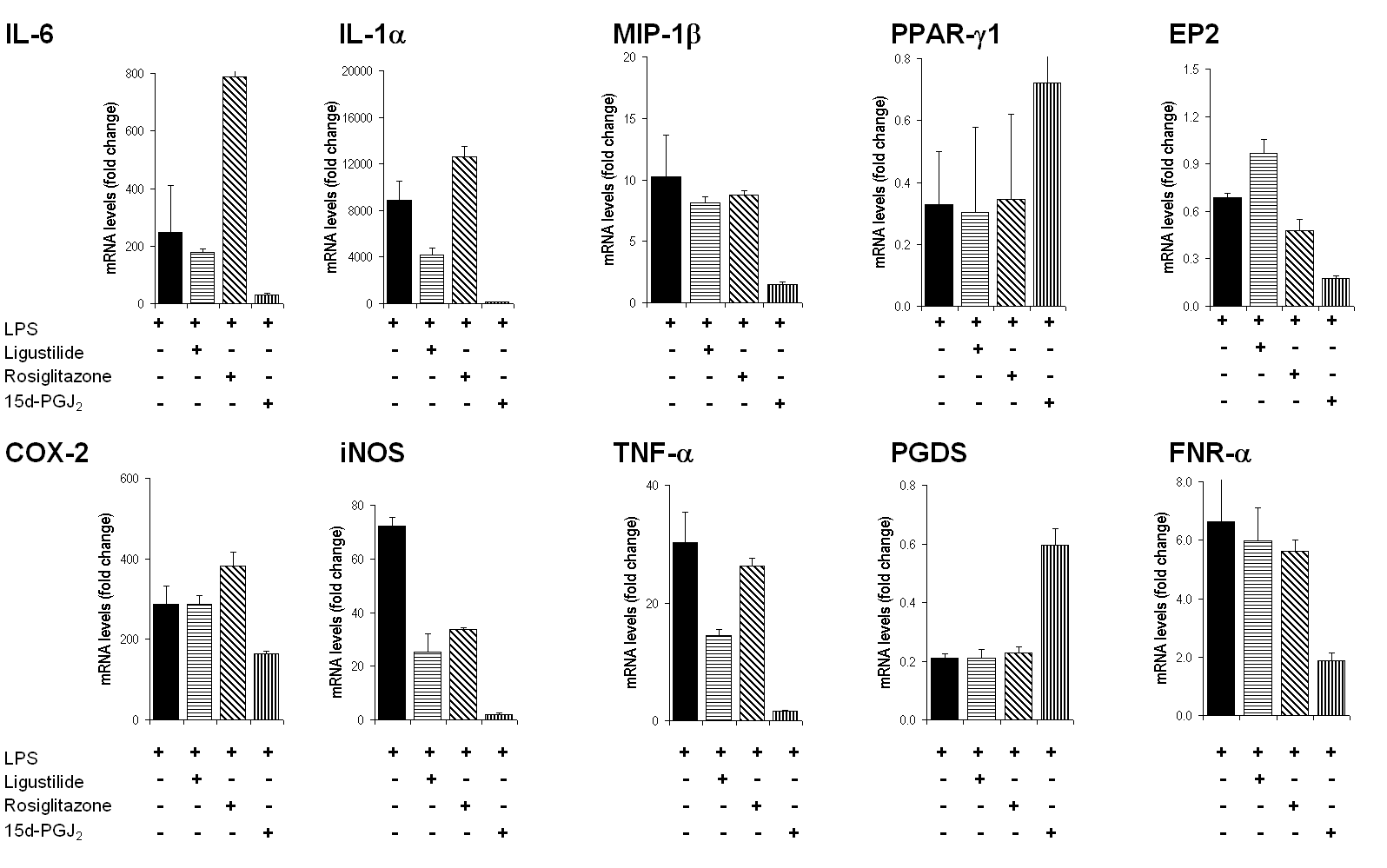

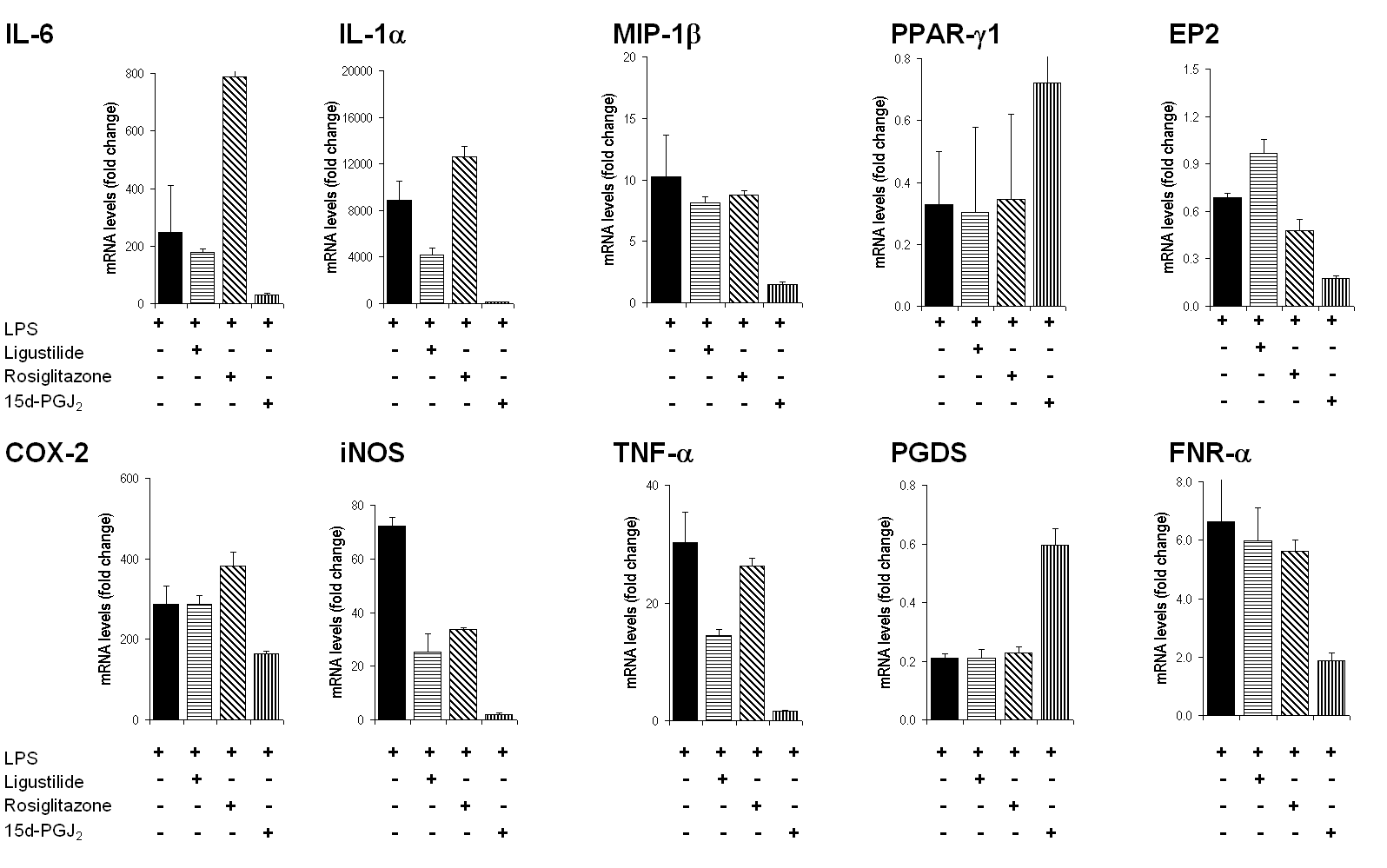

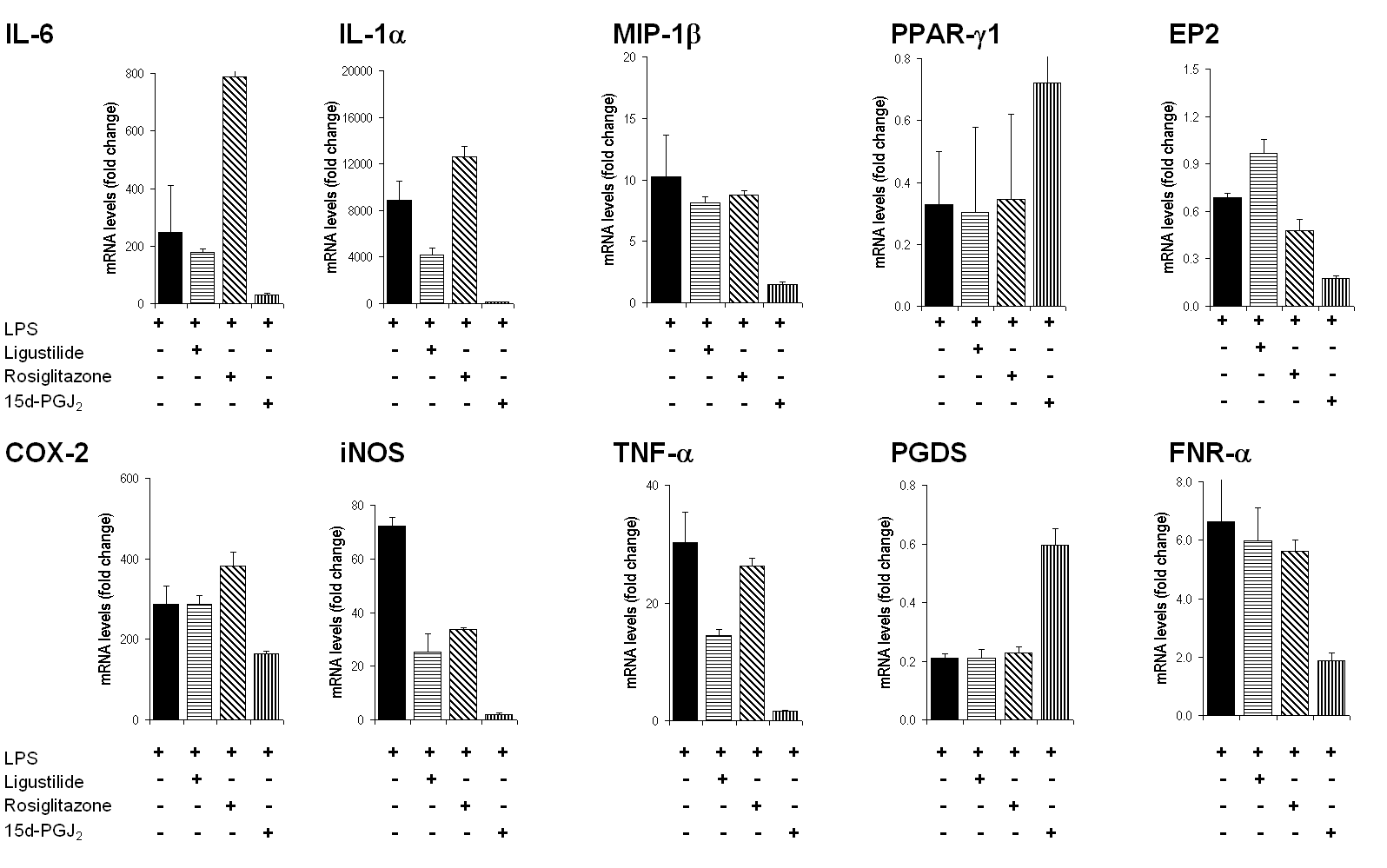


## Table S1:

## *Table S1a: Sequences of human primers and probes (5’ 🡪3’) used for quantitative RT-PCR*

## *Supplementary Table Primers and Probes*

| Gene name | Forward Primer | Reverse Primer | Probe (FAM conjugated) |
| --- | --- | --- | --- |
| PPAR-gamma1 | TCAGAAATGCCTTGCAGTG | TTCTCGGCCTGTGGCATC | TGTCTCATAATGCCATCAGGTTTGGGC |
| IL-1alpha | CTGGAGGCCATCGCCA | TGCTCAGGAAGCTAAAAGGTGC | TGACTCAGAGGAAGAAATCATCAAGCCTAGGTC |
| IL-1beta | ggcctcaaggaaaagaatctgtac | gggatctacactctccagctgtaga | tcctgcgtgttgaaagatgataagccca |
| IL-6 | TGACAAACAAATTCGGTACATCCT | TCTGCCAGTGCCTCTTTGCT | CAGCCCTGAGAAAGGAGACATGTAACAA |
| TNF-alpha | TCGAACCCCGAGTGACAA | AGCTGCCCCTCAGCTTG | CCTGTAGCCCATGTTGTAGCAAACCC |
| COX-2 | gcccttcctcctgtgcc | aatcaggaagctgctttttaccttt | atgattgcccgactcccttgggtgt |
| 5-lipoxygenase | cggcgatgtcgaggttg | tgtgaatttggtcatctcggg | tgagggatggacgcgcaaagttg |
| PDGS | ATTGTGGACACTCTGGATGATTTC | GTGAGCAGCTCATTGAACATCTG | TGGGCAGAGAAAAAGCAAGATGTGAAAGA |
| PGES | TGTACGTGGTGGCCATCATC | CTCAGGGCATCCTCGGG | AGGCTGCGGAAGAAGGCCTTTGC |
| MIP-2 | agtcccccggacccc | gcccattcttgagtgtggc | ctgcgcccaaaccgaagtcat |
| CCL2/MCP-1 | TCGCGAGCTATAGAAGAATCACC | CCTTGGCCACAATGGTCTTG | CAGCAAGTGTCCCAAAGAAGCTGTGATCT |
| CCL5/RANTES | ACCAGTGGCAAGTGCTCCA | GCACACACTTGGCGGTTCTT | CCCAGCAGTCGTCTTTGTCACCCG |
| CXCL8/IL-8 | ACTGACATCTAAGTTCTTTAGCACTCC | GCCTTCCTGATTTCTGCAGC | TGGCAAAACTGCACCTTCACACAG |
| CXCL10/IP-10 | TGAAATTATTCCTGCAAGCCAA | CAGACATCTCTTCTCACCCTTCTTT | GTCCACGTGTTGAGATCATTGCTACAATG |
| 18 S rRNA (housekeeping gene) | CGGCTACCACATCCAAGG | CGGCTACCACATCCAAGG | CGGCTACCACATCCAAGG |

## *Table S1b: Sequences of murine primers and probes (5’ 🡪 3’) used for quantitative RT-PCR*

| Gene name | Forward Primer | Reverse Primer | Probe (FAM conjugated) |
| --- | --- | --- | --- |
| PPAR-gamma1a | gactaccctttactgaaattaccatgg | cacggagaggtccacagagc | tctggcccaccaacttcggaatcag |
| COX-2 | aacatggactcactcagtttgttga | ccactgcttgtacagcaattgg | cagattgctggccgggttgctggggga |
| IL-1alpha | CTGGAGGCCATCGCCA | TGCTCAGGAAGCTAAAAGGTGC | TGACTCAGAGGAAGAAATCATCAAGCCTAGGTC |
| IL-1beta | tgtaatgaaagacggcacacc | ttctttgggtattgcttggga | tccacactctccagctgcaggg |
| IL-6 | ccagaaaccgctatgaagttcc | caccagcatcagtcccaaga | tctgcaagagacttccatccagttgcc |
| IL-12p35 | CAGTCCCGAAACCTGCTG | AGTGCAGGAATAATGTTTCAGTTTT | CTCTGGCCGTCTTCACCATGTCATCTGT |
| iNOS | cagctgggctgtacaaacctt | gaatgtgatgtttgcttcggac | cgggcagcctgtgagacctttga |
| TNF-alpha | atggcccagaccctcaca | ttgctacgacgtgggctaca | tcagatcatcttctcaaaattcgagtgacaagc |
| FNR-alpha | Tccccaaaagaaacttcaggtg | accattgctgccttctgcc | cacagccgtgcagtggacca |
| 5-lipoxgenase | CATGTACCGCCAGCTGCC | TGGTGAACCTCACATGGGC | TGTGCATCCCCTTTTCAAGCTGCTG |
| PGDS | caagccaactcttccatttgg | cgaggctctggtggattgta | atccctgttttggaggtggaaggac |
| PGES | ggcctccagtattacaggagtgac | ccatgtcgttgcggtgg | cagatgtggagcgctgcctcagag |
| CCL4/MIP-1beta | ctcttgctcgtggctgcc | gggagggtcagagccca | tctgtgctccagggttctcagcacc |
| CCL5/RANTES | GCAAGTGCTCCAATCTTGCA | CTTCTCTGGGTTGGCACACA | CGTGTTTGTCACTCGAAGGAACCGC |
| CXCL10/IP-10 | TGAAATTATTCCTGCAAGCCAA | CAGACATCTCTTCTCACCCTTCTTT | GTCCACGTGTTGAGATCATTGCTACAATG |
| MIP-2 | agtcccccggacccc | gcccattcttgagtgtggc | ctgcgcccaaaccgaagtcat |
| MMP9 | CAGGATAAACTGTATGGCTTCTGC | GCCGAGTTGCCCCCA | ACCCGAGTGGACGCGACCGTAG |
| Prostaglandin EP2 receptor | tctggtggtgctcatctgctc | tttcaccacgtttggctgatataa | tccgctcgtggtgcgagtgttc |
| 18S rRNA (housekeeping gene) | CGGCTACCACATCCAAGG | CGGGTCGGGAGTGGGT | TTGCGCGCCTGCTGCCT |

## Table S2:

## Synopsis of effects of ligustilide and 15d-PGJ_2_ on gene expression or protein secretion by (murine) macrophages or peripheral blood leukocytes (human)

| Gene or Protein | Effect of ligustilide | | Effect of 15d-PGJ_2_ | |
| --- | --- | --- | --- | --- |
|  | Murine macrophages | Human PBLs | Murine macrophages | Human PBLs |
| CCL2/MCP-1 | Decreased | Decreased | Decreased | Increased |
| CCL4/MIP-1β | Decreased | Decreased | None | Decreased |
| CCL5/RANTES | Decreased | Increased | None | Increased |
| CXCL8/IL-8 | - | None | - | None |
| CXCL10/IP-10 | - | Decreased | - | Decreased |
| IFN-γ | Decreased | Decreased | Decreased | Decreased |
| TNF-α | Decreased | Decreased | Decreased | None |
| GM-CSF | Decreased | Increased | Decreased | Increased |
| IL-1α | Decreased | - | Decreased | - |
| IL-1β | Decreased | Increased | None | Decreased |
| IL-6 | Decreased | Increased | Decreased | Increased |
| IL-12p70 | Decreased | Decreased | Decreased | Decreased |
|  |  |  |  |  |
